# Resistive Switching and Charge Transport in Laser-Fabricated Graphene Oxide Memristors: A Time Series and Quantum Point Contact Modeling Approach

**DOI:** 10.3390/ma12223734

**Published:** 2019-11-13

**Authors:** N. Rodriguez, D. Maldonado, F. J. Romero, F. J. Alonso, A. M. Aguilera, A. Godoy, F. Jimenez-Molinos, F. G. Ruiz, J. B. Roldan

**Affiliations:** 1 Department of Electronics and Computer Technology, Science Faculty, University of Granada, Av. Fuentenueva s/n, 18071 Granada, Spain; davidmaldonado@correo.ugr.es (D.M.); franromero@ugr.es (F.J.R.); agodoy@ugr.es (A.G.); franruiz@ugr.es (F.G.R.); jroldan@ugr.es (J.B.R.); 2 Pervasive Electronics Advanced Research Laboratory, University of Granada, 18071 Granada, Spain; 3 Department of Statistics and Operations Research, Science Faculty, University of Granada, Av. Fuentenueva s/n, 18071 Granada, Spain; falonso@ugr.es (F.J.A.); aaguiler@ugr.es (A.M.A.)

**Keywords:** memristor, RRAM, variability, time series modeling, autocovariance, graphene oxide, laser

## Abstract

This work investigates the sources of resistive switching (RS) in recently reported laser-fabricated graphene oxide memristors by means of two numerical analysis tools linked to the Time Series Statistical Analysis and the use of the Quantum Point Contact Conduction model. The application of both numerical procedures points to the existence of a filament connecting the electrodes that may be interrupted at a precise point within the conductive path, resulting in resistive switching phenomena. These results support the existing model attributing the memristance of laser-fabricated graphene oxide memristors to the modification of a conductive path stoichiometry inside the graphene oxide.

## 1. Introduction

Memristors have shown great potential in the context of neuromorphic circuits. Their operation, based on resistance modulation by means of ion transport and redox reactions, leads to the creation of regions of different conductivity mimicking neuronal synapses in a coherent and natural manner. Consequently, memristors are of most interest for the fabrication of optimized hardware that aims to design and implement artificial neural networks [1,2,3]. This potential, along with their intrinsic facet of non-volatility, poses the set of features needed by memristors to become the cornerstone for computation schemes beyond of the classical von Neumann paradigm, such as neuromorphic computing. This new focus will be essential to push forward the artificial intelligence challenges that the industry is facing currently [2,3].

From a more general perspective, the outstanding features of memristors make them also suitable for applications that run through non-volatile memories, Internet of Things (IoT) devices, 5G, etc. Among their promising characteristics, the following can be highlighted: fast read/write times for the set and reset processes, low power consumption, scalability and CMOS technology compatibility among others [3,4,5,6,7].

The physics behind memristors is strongly dependent on the materials employed and the details of their fabrication process. In this respect, there is a plethora of recent experimental, modeling and simulation studies on technologies that make use of transition metal oxides as the switching dielectric [4,5,8,9,10,11,12,13,14,15]. However, in the field of memristors based on 2D materials, the amount of studies and published manuscripts is much lower. In this context, the difficulties related to the creation of high quality metal contacts, the purity of the materials and the fabrication details pose extra difficulties for dealing with all of the facets of the study of these devices, and in particular, in regards to the physical simulation and modeling.

In the 2D material memristors landscape, there are h-BN based devices, memristors with a different number of graphene layers or other 2D materials that are employed for oxygen ion scavenging and other particular purposes [3,16,17]. Among all the 2D materials-based contenders, the laser fabrication of memristors based on graphene oxide (GO) was recently introduced [18]. GO is a highly functionalized form of polycrystalline nanographene that is decorated with oxygen-containing groups [19]. The use of GO as a memristive material takes advantage of its inherent 2D materials potential with respect to conduction and structural flexibility properties while simultaneously including its non-volatility and electrical plasticity [20], as expected in ideal memristors [21].

The implementation of a laser-assisted fabrication protocol provides the device with several attractive features for its potential industrial implementation: (i) the fabrication process is very simple, comprising a limited number of steps; (ii) there is no need for lithographic masks since the laser itself defines the geometry of the memristor; (iii) the devices do not require scarce or hazardous materials for their fabrication; (iv) the resistive switching behavior originates in the GO (and not in the electrodes) adding versatility from the contacting electrodes perspective and (v) the supporting substrate can be selected with versatility from a rigid surface to flexible polymers for conformal integration.

The novelty of the devices employed here results in a lack of studies linked to their resistive switching features, both from the physical modeling and experimental viewpoint. Therefore, the physics lying behind their operation has only had its surface scratched [18]. In this work, we intend to tackle this issue making use of well-established numerical techniques previously developed for more “conventional” memristors that are developed with 3D stacks of transitions metal oxides [13,15,22,23]. Therefore, in this manuscript, we specifically deal with the characterization and analysis of resistive switching processes and charge conduction in laser-fabricated graphene oxide (GO) memristors [18] from a statistical perspective. We do not focus this study on the digital performance of the devices; we consider instead their conductance variation in an analogic manner, as it is the proper approach for neuromorphic applications.

The device variability has also been considered in this study, specifically by using Time Series Statistical Analysis (TSSA) [24,25,26,27]. From the statistical viewpoint, information can be extracted that is related to the correlation of successive RS cycles and the inherent stochasticity of RS memristors operation. The quantum properties of conduction along the conductive filaments that short the electrodes have been scrutinized by means of the Quantum Point Contact (QPC) model as described in [15,22].

Therefore, the outline of this work is as follows: the fabricated devices and measurement process are described in Section 2, and the numerical procedure, the main results and the discussion are explained in Section 3. Finally, the conclusions are given in Section 4.

## 2. Device Fabrication and Measurement 

**Figure 1:**
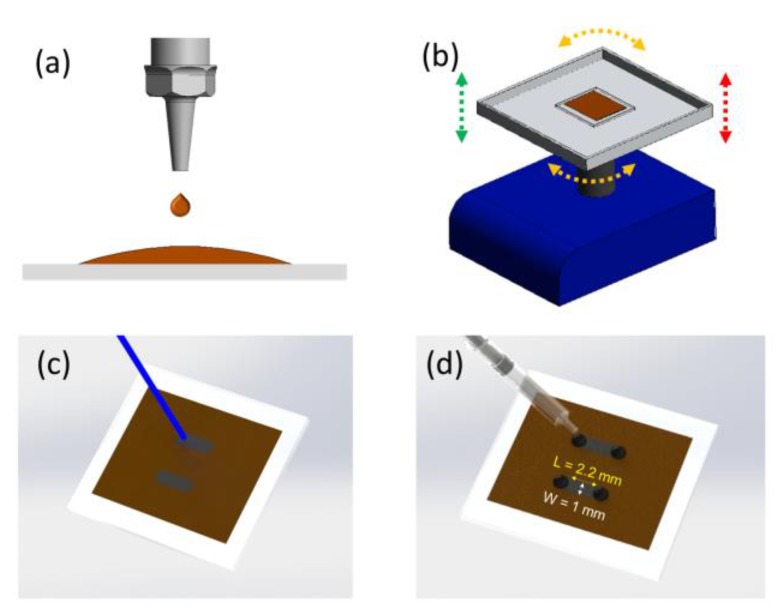
Schematic representation of the fabrication steps for graphene oxide memristors produced by laser. Graphene Oxide colloid is drop-casted on a PET substrate (**a**) and left 48 h on a 3D shaker for water evaporation (**b**). Then the laser diode is applied (70 mW) to partially reduce the GO resulting in the memristive structures (**c**). Finally, electrical contacts are created by depositing microdrops of organic bare conductive paint (**d**).

The memristors fabricated for this study are fully based on the process described in [18] and summarized in Figure 1. The raw precursor material is a graphene oxide colloid (4 mg/mL) prepared following a modified version of Hummers and Offerman’s method [28]. The GO colloid is deposited by drop-casting onto a PET (Polyethylene terephthalate, 3 M) film (0.5 mL/cm^2^) and left on a 3D-shaker for 48 h until the water has completely evaporated (293 K, RH 50%). The CNC-driven laser is then applied in a rectangular pattern with the precise power that reduces the GO at the point where memristance is manifested (P_laser_ ~ 70 mW, λ = 405 nm) [18]. After the laser treatment, the volume of the reduced GO increases; the height difference between the GO film and the laser-treated GO is ~10 µm, determined using a DekTak XT profilometer from Bruker (Bruker Corporation, MA, USA). The devices were contacted using micro drops of conductive carbon-based paste (Bare Conductive Electric Paint, London, UK).

**Figure 2:**
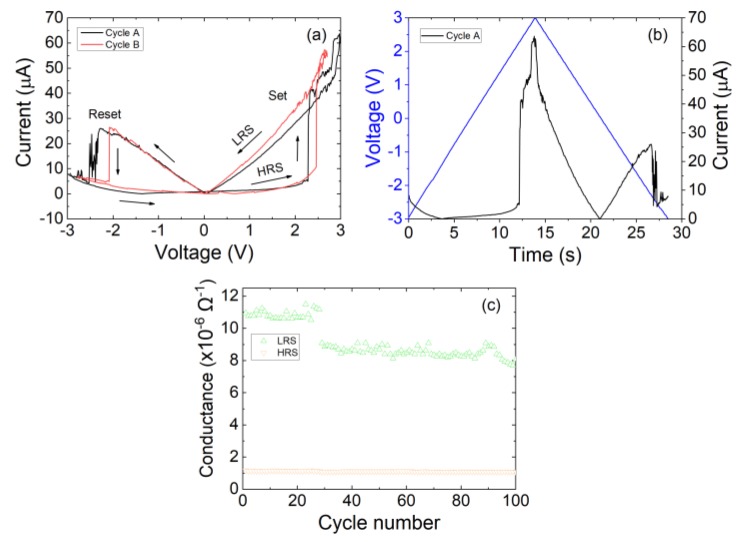
(**a**) Experimental current versus voltage for two different cycles within a resistive switching series. A ramped voltage with step of 10 mV was employed in the measurement process. (**b**) Voltage and current versus time for the cycle A shown previously. (**c**) Conductance values obtained during device cycling with limited compliance current [18]. The resistance was extracted in the range [−1,1] V of the current–voltage characteristics.

The electrical measurement experiments were performed with the support of a two-channel Keysight^®^ B2902A (Keysight Technologies, Inc., CA, USA) precision source-measurement unit controlled by Easy-Expert^®^ software (version 6.2.1927.7790, CA, USA). Figure 2a presents measured current–voltage characteristics showing two consecutive voltage cycles extracted from an L = 2.2 mm, W = 1 mm laser-fabricated graphene oxide memristor. These curves reveal the characteristic fingerprint of a memristor device that is determined by a pinched hysteresis loop closed in the origin of the current–voltage axis [29]. Figure 2b depicts the time evolution of the current when a −3 to 3 V symmetric voltage ramp is applied, illustrating the fast and abrupt transitions of the resistance.

Figure 2c shows the device conductance extracted under successive device cycling from a laser-fabricated GO memristor. These measurements constitute the input of the Time Series Statistical Analysis discussed in Section 3. To avoid resistive switching degradation of the device, the current is limited to 20 µA [18]. As observed, the Low Resistance State (LRS) conductance presents a monotonic derivative, whereas the High Resistance State (HRS) conductance remains stable with cycling. The reader can notice the small conductance jump at cycle 28. This phenomenon is attributed to the defective nature of GO, which is heavily decorated with oxygen, hydroxyl and epoxy groups. Spontaneous movements of functional groups along the conductive path yields to local modification of the stoichiometry of the sample and, therefore, to the modification of its conductance [19]. Further structural and electrical details of Laser-Fabricated Graphene Oxide Memristors can be found in reference [18], including spectroscopic characterization, retention time and variability. The electrical results (average HRS/LRS ratio, 6; retention time, 10^4^ s; endurance, 10^2^ cycles [18]) can be considered to be promising given the early stage of development of this technology, and they are expected to become more attractive once advanced laser lithography tools are employed for the development of GO laser-fabricated memristors.

## 3. Numerical Analysis of Charge Conduction and Resistive Switching Mechanisms, Results and Discussion

### 3.1. Time Series Statistical Analysis (TSSA)

**Figure 3:**
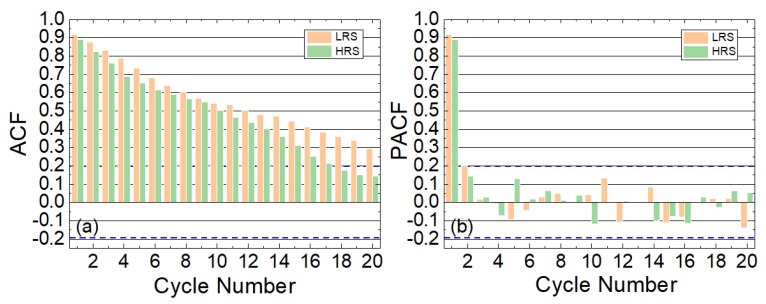
(**a**) ACF and (**b**) PACF versus cycle lag for the inverse of the values shown in Figure 2c. These functions show the ACF and PACFs versus cycle number that represent the distance apart in cycles within a RS series, see Reference [24]. The ACF and PACF minimum threshold bounds for the devices under study are ±0.195 for both plots (see the supplementary information for the information linked to the calculation of these threshold bounds), shown with dashed lines. We have considered 100 cycles in our series; this is a reasonable number to extract information on the correlation between the data and to extract a TSSA model.

The TSSA has been employed to characterize the statistical features of the device operation variables through a long RS series [24]. In particular, the resistances in the LRS and HRS have been studied. The Autocorrelation (ACF) and Partial Autocorrelation functions (PACFs) have been calculated and represented in Figure 3 (see also Supplementary Materials). As can be observed, the degree of correlation between the measurements of previous cycles is very high with respect to other technologies (see, for instance, Reference [24] for other technologies with transition metal oxides as a dielectric).

It can be concluded that to obtain these results, the high conductivity region does not change much between different cycles; this feature is the main source of the correlation. This fact leads us to assume a filamentary-like conduction mechanism where a channel of high conductivity region is formed after a set process that shorts the electrodes. In addition, the high correlation suggests that the high conductivity path does not change much between cycles, keeping unaltered the main conduction properties. It is reasonable to assume that it is just a narrow region that changes in between two larger high conductivity regions that remain mostly unaltered. This narrowing is modified leading to the creation of a fully-formed high conduction path that shorts the electrodes or that isolates them in case the path is ruptured, leading to two large virtual electrodes (filaments remnants connected to the electrodes [6]).

We have employed TSSA to analytically describe the dependencies of the LRS and HRS resistances on previous cycles throughout the complete RS series (see in the Supplementary Material a summary of the steps needed to develop a TSSA model). The general expression employed was based on an Autoregressive (AR) approach [24], as seen in Equation (1):

R_LRS/HRS(t)_ = Φ_1_ × R_LRS/HRS(t-1)_ + Φ_2_ × R_LRS/HRS(t-2)_ + … + Φ_p_ × R_LRS/HRS(t-p)_ + ε_t_ (1)

where *t* stands for the cycle number within a long resistive switching series. In this modeling technique, the order (*p*) is linked to the physics governing RS process in these devices. No previous knowledge is assumed to extract the information from experimental data because the underlying technology details and physics mechanisms are “hidden” in the RS data collected. The TSSA models are empirical and determine the weights set (Φ_1_, ..., Φ_p_), and the model order is determined by *p*. The term ε_t_ is a residual that accounts for the model error (the difference between the measured and the modeled value). In this respect, we focus here on the statistical information of the measured data without any previous assumption linked to the underlying physics.

The resistance at the LRS can be modeled with an AR(2) approach, as seen in Equation (2).

R_LRS(t)_ = 4936.018 + 0.7306 × R_LRS(t-1)_ + 0.229 × R_LRS(t-2)_ + ε_t_. (2)

The HRS resistance works well with an AR(1), as described in Equation (3).

R_HRS(t)_ = 69955.16 + 0.9236 × R_HRS(t-1)_ + ε_t_. (3)

The time series residuals that are left after a comparison with the experimental data show a white noise behavior; therefore, we can conclude that all the statistical information is included in the models described in Equations (2) and (3). It is important to highlight at this point that TSSA is an ideal tool used to analyze data in a series (such as a RS series); in this respect, it works well for cycle-to-cycle variability analysis if we consider parameters such as the set and reset voltages or LRS/HRS device resistances.

### 3.2. Quantum Point Contact Modeled Conduction

**Figure 4:**
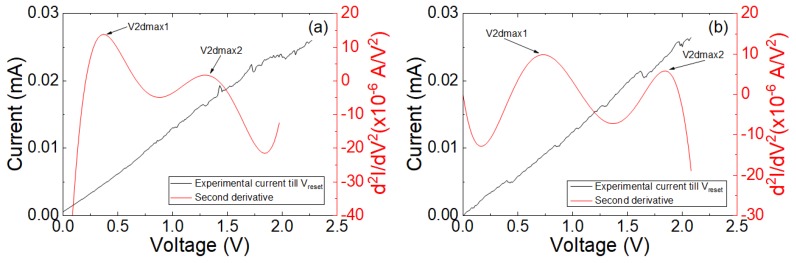
Experimental current versus applied voltage in the devices under study including the second derivative of the current versus voltage for cycle A (**a**) and cycle B (**b**) shown in Figure 2a. A pattern in agreement with the QPC model is seen in [22].

An analysis of the I–V curves in terms of second derivative dependencies has been performed following [22]. In this respect, it is important to highlight that a screening procedure was developed in [22] to detect charge conduction features that can be modeled with the QPC model. The results are shown in Figure 4.

**Figure 5:**
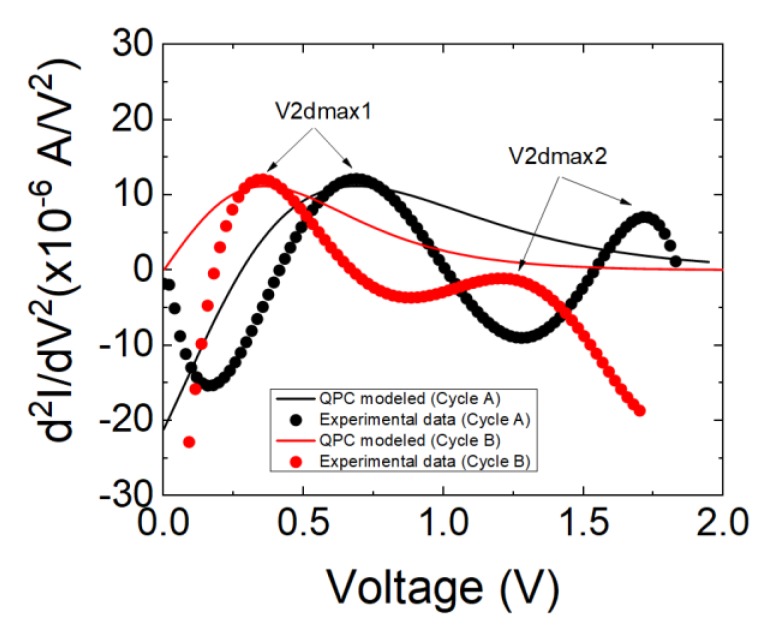
Second derivative of the experimental current (symbols) versus voltage in the device under study for the two reset curves shown in Figure 2. The analytically calculated QPC modeled current second derivative (solid lines) is also shown. The QPC model parameters employed for cycle A are the following: α = 6.5 (eV)^−1^; β = 0.4; Φ = 0.13 eV; N = 1; and for cycle B: α = 7.5(eV)^−1^; β = 0.5; Φ = 0.055 eV; N = 1.

The characteristic one or two maxima in the current second derivative are seen in these devices. Following previous results [22], this behavior could be regarded as a footprint of the existence of QPC conduction. However, the fitting of the second derivative leads to an N parameter (number of channels in the QPC model [22]) lower than the unity, which is inconsistent with the QPC model. In this respect, a new representation is obtained assuming a series resistance of 5000 Ω (second numerical derivative of the corrected current, I, taking into account the series resistance is shown in Figure 5). This series resistance is reasonable considering the device resistance both at LRS and HRS, see Figure 2c. In this manner, the voltage on the constriction that leads to quantum effects can be obtained accurately.

In both cases, there is only one channel for charge conduction, and this result corresponds to a low dimensional high conductivity region. Also, a low energy barrier is observed, suggesting an almost ohmic charge conduction regime, although in a low conductivity regime when compared with conventional memristors based on transition metal oxides.

The previous results support the existing model that attributes resistive switching in laser-reduced GO to the non-uniformity in the number and location of functional groups that create nanometric-size regions of different conductance [18]. The sp^2^ regions present high-conductivity but they are interrupted by low-conductivity sp^3^ domains at a nanoscale level that are responsible for a low current flow [30,31]. At certain locations within the structure, under the action of the voltage bias in the HRS, large electrostatic potential gradients are created in the nanometric-size low-conductivity regions, resulting in large localized electric fields. Assisted by Joule heating effects, these electric fields can trigger the drift of oxygen and oxygen-containing groups due to the low migration barrier in GO [32,33]. The group migration at a specific point within the structure establishes a continuity path of sp^2^ domains, which was previously impeded by a nanometric sp^3^ domain (quantum point contact as identified in this work) and leads to a LRS [18]. Finally, it is worth mentioning that the findings in this work, disclosing the filamentary nature of the conduction in laser fabricated GO memristors, open the path for scaling the devices down by using high precision laser scribing systems.

## 4. Conclusions

The origins of resistive switching in recently introduced laser-fabricated graphene oxide memristors have been studied by using statistical and numerical analysis tools. Time Series Statistical Analysis applied to the high and low resistance states of the devices has shown high correlation that supports the model of the formation of a conductive filament as the main source of the device internal resistance switching. Furthermore, the quantum point contact conduction method has pointed to the existence of a quantized point of conduction, which is formed and destroyed, connecting the electrodes by means of a conductive path. These results underpin the existing theory that attributes the memristance in GO to the formation of a highly reduced path in which stoichiometry is modified at a precise point leading to the resistive switching.

## Supplementary Materials

The following are available online at https://www.mdpi.com/1996-1944/12/22/3734/s1.
