# Supplementary material for: Resistive Switching and Charge Transport in Laser-Fabricated Graphene Oxide Memristors: A Time Series and Quantum Point Contact Modeling Approach
Source: Materials (Basel). 2019 Nov 13;12(22):3734. doi: 10.3390/ma12223734 (PMC6888125; doi:10.3390/ma12223734)
Supplement: Supplementary file 1 [file materials-12-03734-s001.pdf]

materials

MDPI

Resistive Switching and Charge Transport in Laser-Fabricated Graphene Oxide Memristors: A Time Series and Quantum Point Contact Modeling Approach

N. Rodriguez [1,2,*], D. Maldonado [1], F.J. Romero [1,2], F.J. Alonso [3], A.M. Aguilera [3], A. Godoy [1,2], F. Jimenez-Molinos [1], F.G. Ruiz [1,2] and J.B. Roldan [1]

[1] Department of Electronics and Computer Technology, Science Faculty, University of Granada, Av. Fuentenueva s/n, 18071 Granada, Spain; davidmaldonado@correo.ugr.es (D.M.); franromero@ugr.es (F.J.R.); agodoy@ugr.es (A.G.); jmolinos@ugr.es (F.J.M.); franruiz@ugr.es (F.G.R.); jroldan@ugr.es (J.B.R.)
[2] Pervasive Electronics Advanced Research Laboratory, University of Granada, 18071 Granada, Spain
[3] Department of Statistics and Operations Research, Science Faculty, University of Granada, Av. Fuentenueva s/n, 18071 Granada, Spain; falonso@ugr.es (F.J.A.); aaguiler@ugr.es (A.M.A.)
* Correspondence: noel@ugr.es

The Autocorrelation Function (ACF) is calculated along with the Partial Autocorrelation Function (PACF) to determine the coefficients of the models in the context of the Time Series Analysis (TSA) [1,2]. The ACF helps to visualize the statistical information related to the correlation between the data of successive cycles within a resistive switching series. The mean value (Equation (A1)) of the data is needed for the ACF determination:

$$E(R_{LRS_t}) = E(R_{LRS_{t-k}}) = \mu \tag{A1}$$

where R_{LRS_t} is the resistance in the low resistance state in the current cycle of a Resistive Switching (RS) series, and $R_{LRS_{t-k}}$ is the resistance in the low resistance state lagged *k* cycles, i.e., the resistance in the low resistance state measured *k* cycles before the current cycle *t* in the RS series.

As the variance is also required for the ACF determination, it can be obtained as shown in Equation (A2):

$$\mathrm{Var}\,(R_{LRS_t}) = \mathrm{E}\left[(R_{LRS_t} - \mu)^2\right] = \mathrm{Var}(R_{LRS_{t-k}}) = \sigma. \tag{A2}$$

The autocovariance is obtained as follows:

$$\gamma(\mathrm{k}) = \mathrm{Cov}(R_{LRS_{t-k}}, R_{LRS_t}) = \mathrm{E}\left[(R_{LRS_{t-k}} - \mu)(R_{LRS_t} - \mu)\right]. \tag{A3}$$

Hence, with all this information, the ACF can be calculated as shown in Equation (A4), and it can be employed to estimate the degree of dependence among the data (resistance in the low resistance state in this case) of different cycles. Precisely, for our case, the ACF summarizes how correlated the data lagged *k* cycles are. If we take into consideration that $\gamma(0)$ stands for the variance, Equation (A4) can be simplified.

$$\rho(k) = Cor(R_{LRS_{t-k}}, R_{LRS_t}) = \frac{\mathrm{Cov}(R_{LRS_{t-k}}, R_{LRS_t})}{\sqrt{\mathrm{Var}(R_{LRS_t})\mathrm{Var}(R_{LRS_{t-k}})}} = \frac{\gamma(k)}{\gamma(0)}. \tag{A4}$$

TIME SERIES MODEL

The formulation of an AutoRegressive Moving Average (ARMA) time series model, ARMA(*p*,*q*), is given as follows,

$$R_{LRS_t} = \emptyset_0 + \emptyset_1 R_{LRS_{t-1}} + \cdots + \emptyset_p R_{LRS_{t-p}} - \theta_1 \varepsilon_{t-1} + \cdots - \theta_q \varepsilon_{t-q} \tag{A5}$$

where R_{LRS_t} is the modeled low resistance states of the device in the current cycle of an RS series, $R_{LRS_{t-k}}$ is the modeled device resistances lagged *k* cycles (namely, the *k* device resistance values of the previous RS cycles) and ε_{t-k} stands for the residuals found in the modeling process from earlier cycles (differences between the modeled and measured data in each cycle) with $\emptyset_i$*(i=1, …, p)* and θ_j*(j=1, …, q)* being the unknown regression coefficients to be estimated. In time series methodology, ε_t is also considered; however, in our case, we assume that it is not included in the formulation as in

Reference [3]. Therefore, the actual device resistance in the LRS can be calculated by means of two linear polynomials: one for the autoregressive part (AR, in this case a linear function of some of device resistance past values), and the other for the moving average part (MA, a linear combination of the past residuals). p and q indicate the orders of the autoregressive and the moving average terms, respectively.

ARMA modeling can be carried out in five stages: stationarity check, model identification, parameter estimation, validation and prediction. Each one is briefly described as follows:

1. Stationarity Check

We must ensure that the time series is stationary. This means that no change in the mean and the variance is observed over time. These values have to be distributed around a value (the mean) and the fluctuations above and under this value have to be similar in all the series range (the series data have to be within a constant interval). In addition, the sample ACF must tail off near zero after a few lags. Some transformations of the original variable to be modeled (logarithm, differentiation…) can be employed to convert a non-stationary series to a stationary one. This process would be performed over the transformed series, and a final reverse transformation would be required to recover the original variable.

2. Model Identification

We need to estimate the values of p and q and the order of the AR and MA polynomials, respectively. The ACF and PACF must be compared to the corresponding theoretical ACF and PACF for several ARMA models.

The PACF evaluates the correlation between two variables separated by k periods in the series if the dependency due to the intermediate lags is eliminated. That is,

$$\begin{aligned}\pi(\mathrm{k}) &= \mathrm{Cor}\left(R_{LRS_{t-k}}, R_{LRS_t} \middle| R_{LRS_{t-1}}, R_{LRS_{t-2}}, \ldots, R_{LRS_{t-k+1}}\right) \\ &= \mathrm{Cor}\left(R_{LRS_{t-k}} - \widehat{\mathrm{R}}_{\mathrm{LRS}_{\mathrm{t-k}}}, \mathrm{R}_{\mathrm{LRS}_{\mathrm{t}}} - \hat{R}_{\mathrm{LRS}_{\mathrm{t}}}\right)\end{aligned} \quad \text{(A6)}$$

where $\hat{R}_{\mathrm{LRS}_{\mathrm{t}}}$ is the estimated value by the linear regression model of R_{LRS_t} on the lagged variables $R_{LRS_{t-1}}, R_{LRS_{t-2}}, \ldots, \mathrm{R}_{\mathrm{LRS}_{\mathrm{t-k+1}}}$. $\pi(\mathrm{k})$ can be held by making the following regression

$$R_{LRS_t} = \varphi_{k1} R_{LRS_{t-1}} + \varphi_{k2} R_{LRS_{t-2}} + \cdots + \varphi_{k,k-1} R_{LRS_{t-k+1}} + \varphi_{kk} R_{LRS_{t-k}}.$$

In this context, $\pi(\mathrm{k}) = \varphi_{kk}$. Thus, the PACF is an essential tool for detecting the order p of the AR (p) models. If the PACF is zero after lag p, then the data structure is well modeled using the p previous observed values. Consequently, an AR model is sufficient. The regression term number (p) is linked to the last significant value with respect to the threshold bounds in the PACF.

If the ACF is zero after lag q, a MA model is used. The order is given by the last significant term in the ACF function. If both functions, the sample ACF and PACF, tail off to zero, then the model needs autoregressive and moving average terms.

The sample partial autocorrelation $\hat{\pi}(\mathrm{k})$ at a certain lag k is considered to be zero if they do not exceed the significance bound (threshold) given by,

$$\pm \frac{1.96}{\sqrt{\mathrm{n}}} \quad \text{(A7)}$$

where n is the number of observed data (number of cycles in the RS series in our case) [2].

The autocorrelation $\hat{\rho}(\mathrm{k})$ at a certain lag k is considered to be zero if it does not exceed the significance bound (threshold) given by [2],

$$\pm \frac{1.96}{\sqrt{\mathrm{n}}} \sqrt{1 + 2\widehat{\rho^2}(1) + \cdots + 2\,\widehat{\rho^2}(\mathrm{k}-1)}. \quad \text{(A8)}$$

If there is not correlation structure, this formula is equivalent to Equation (A7).

3. Parameter Estimation

Once an ARMA model is selected, the non-linear least square method is used for the calculation of the parameters. This technique also allows us to determine the residuals, ε_t, for all t.

4. Validation

We must check that the different residuals are not correlated. The residuals (ε_j) ACF and PACF are obtained and plotted, the data have to be within the threshold bounds (Equations (A7) and (A8)).

5. Prediction

Once the best model is achieved, the predictions of future values within the time series calculation scheme can be made.

References

1. Bisgaard, S.; Kulahci, M. Time Series Analysis and Forecasting by Example, Wiley: NJ, USA, 2011.
2. Brockwell, P.J.; Davis, R.A. *Introduction to Time Series and Forecasting*, 2ed., Springer: New York, NY, USA, 2002.
3. Roldán, J.B.; Alonso F.J.; Aguilera A.M.; Maldonado, D.; Lanza, M. Time series statistical analysis: A powerful tool to evaluate the variability of resistive switching memories. *J. Appl. Phys*. **2019**, *125*, 174504, doi:10.1063/1.5079409.

© 2019 by the authors. Submitted for possible open access publication under the terms and conditions of the Creative Commons Attribution (CC BY) license (http://creativecommons.org/licenses/by/4.0/).
